# Coherence and divergence in autonomic‐subjective affective space

**DOI:** 10.1111/psyp.14262

**Published:** 2023-02-05

**Authors:** Hélio Clemente José Cuve, Joseph Harper, Caroline Catmur, Geoffrey Bird

**Affiliations:** ^1^ Department of Experimental Psychology University of Oxford Oxford UK; ^2^ School of Psychological Science University of Bristol Bristol UK; ^3^ Department of Psychology, Institute of Psychiatry, Psychology & Neuroscience King's College London London UK; ^4^ School of Psychology University of Birmingham Birmingham UK

**Keywords:** autonomic, electrodermal, emotion, heart rate, multivariate, pupilometry

## Abstract

A central tenet of many theories of emotion is that emotional states are accompanied by distinct patterns of autonomic activity. However, experimental studies of coherence between subjective and autonomic responses during emotional states provide little evidence of coherence. Crucially, previous studies investigating coherence have either adopted univariate approaches or made limited use of multivariate analytic approaches by investigating subjective and autonomic responses separately. The current study addressed this question using a multivariate dimensional approach to build a common autonomic‐subjective affective space incorporating subjective responses and three different autonomic signals (heart rate, skin conductance response, and pupil diameter), measured during an emotion‐inducing task, in 51 participants. Results showed that autonomic and subjective responses could be adequately described in a two‐dimensional affective space. The first dimension included contributions from subjective and autonomic responses, indicating coherence, while contributions to the second dimension were almost exclusively of autonomic covariance. Thus, while there was a degree of coherence between autonomic and subjective emotional responses, there was substantial structure in autonomic responses that did not covary with subjective emotional experience. This study, therefore, contributes new insights into the relationship between subjective and autonomic emotional responses, and provides a framework for future multimodal emotion research, enabling both hypothesis‐ and data‐driven testing.

## Abstract 2

Whether our subjective emotions are accompanied by distinct physiological states is hotly debated. Using a novel multivariate analysis enabling physiological data and subjective experience to be analysed within the same dimensional structure, we find evidence for both coherence between physiological markers of autonomic state and subjective experience, and evidence for a substantial degree of divergence.

## 1 INTRODUCTION

At the heart of most, if not all, mainstream theories of emotion is the idea that emotions involve mobilization of the autonomic nervous system and a change in subjective feeling states. The extent to which subjective feelings and autonomic responses are coordinated in emotional states, however, has remained a topic of fierce debate. While some have claimed the existence of highly specific physiological responses which are mobilized by (or constitute) basic emotions that fulfill adaptive goals (Ekman, 1992; James, 1948; Stemmler, 2004), others have argued that the physiological correlates of emotional states are undifferentiated (Barrett, 2006a; Canon, 1927), and, at most, are explained by more general affective dimensions such as valence and arousal.

In principle, this debate should be relatively easy to resolve – one should be able to measure the physiological correlates of subjective emotional states in order to determine their specificity. Indeed, as documented in many reviews (Cacioppo et al., 2008; Kreibig, 2010; Siegel et al., 2018), this approach has been taken in several studies, including those aiming to identify atypical physiological correlates of emotion in various clinical conditions (Blair, 1999; Lang et al., 1998), or to inform new developments in affective computing (D'Mello et al., 2018; Picard, 2003).

Several markers of autonomic nervous system (ANS) activity have been used to measure physiological correlates of emotional states, with the most common being skin conductance and heart rate (Cacioppo et al., 2008; Kreibig, 2010), with pupil diameter (dilation/constriction) becoming increasingly popular (Finke et al., 2021; Lang & Bradley, 2010). Two features of studies in this area are striking. The first is that different physiological signals are used interchangeably across studies as markers of ANS activity and indicators of emotional response. For example, skin conductance, heart rate, and pupil size are used interchangeably as markers of the physiological arousal associated with emotional responses (Finke et al., 2021; Kreibig, 2010; Lang & Bradley, 2010; Norman et al., 2016; Siegel et al., 2018).

Implicit in the interchangeable use of physiological markers of ANS state during emotion is the assumption of coherence – both between different physiological markers and between physiological markers and subjective feeling states. However, whether this assumption is justified is not clear: for example, while many autonomic signals share some similarities in their dynamic properties (e.g., relatively slow response profiles), they are also controlled in part by different physiological mechanisms (Bradley et al., 2008; Dawson et al., 2016; Mathôt, 2018; Tranel & Damasio, 1994). Different neuronal populations and discrete functional pathways are involved in controlling different sympathetic organs like the skin and heart, and in mediating efferent signaling from the central nervous system (CNS) to the ANS, and afferent signaling from the ANS to CNS (Folkow, 2000; Jänig & McLachlan, 1992). For example, in contrast to sudomotor organs, the heart is dually innervated by both sympathetic and parasympathetic branches of the autonomic system (Berntson et al., 1991; Leuchs et al., 2019), which results in a more complex activity profile than that seen in sudomotor activity. These factors mean that the ANS state data captured by different autonomic signals (e.g., skin conductance, heart rate, and pupil diameter) often differ in terms of their phasic properties, habituation, rate of change, lability, and recovery profiles (Bacigalupo & Luck, 2018; Berntson et al., 1991; Bradley et al., 1993; Leuchs et al., 2019; Snowden et al., 2016). It is perhaps therefore unsurprising, but problematic, that in the few studies to record multiple physiological signals in response to emotion, the correlation between different signals is often modest, and significant only at a group level, rather than within participants (e.g., Bradley et al., 2008; Cacioppo et al., 2008; Kuzinas et al., 2016; Wang et al., 2018).

The rarity of studies measuring physiological responses to emotion in more than one modality is the second striking feature of studies looking to assess the coherence of the physiological and subjective properties of emotional states. To our knowledge, there are only a handful of studies that attempt to characterize physiological responses to emotion using multiple physiological signals (e.g., heart rate, skin conductance, and pupil diameter) in a multivariate manner (e.g., Christie & Friedman, 2004; Kim & Wedell, 2016; Stemmler, 2004). Given the demonstrated differences in the neural correlates and response properties of different signals as detailed above, it is possible that the pattern of responses across physiological signals may be more diagnostic of emotional states than any of the constituent signals alone (Berntson et al., 1991; Friedman et al., 2014; Siegel et al., 2018; Stemmler, 2004). Indeed, different multivariate, but not univariate, patterns of physiological responses during emotional states may explain why the univariate literature on this topic is characterized by such mixed results. For example, while some studies have shown that pupil changes and skin conductance responses track subjective arousal regardless of valence (Bradley et al., 2008), others have shown distinct or asymmetric response profiles for positive and negative emotional experiences (Babiker et al., 2013; Vasquez‐Rosati et al., 2017). Similarly, while negative affective states such as anxiety have been demonstrated to raise heart rate, other studies have reported heart‐rate deceleration as a physiological response to increasingly arousing negative emotion (Bradley & Lang, 2000; Brouwer et al., 2013; Ritz et al., 2005).

Multivariate analysis of multiple physiological signals has the potential to determine that emotional mobilization of physiological systems may best be characterized by patterns of responses across physiological signals. Multivariate data‐driven techniques could identify dimensions or clusters that indicate coherence between different markers of autonomic response, and between autonomic and subjective emotional responses. An advantage of such an approach is that multivariate physiological response profiles for emotion can be identified based on autonomic and subjective variables with limited assumptions about how many dimensions are needed to represent the response, or whether (and how many) emotional categories exist – which are topics of fierce debate among emotion theorists and are thought to drive at least some of the disparate conclusions regarding the extent of autonomic and subjective coherence in emotion (Kreibig, 2010; Levenson, 2014; Norman et al., 2016; Siegel et al., 2018).

The current study addressed these issues in two ways. First, it investigated coherence between autonomic and subjective responses by modeling trial‐by‐trial fluctuations in autonomic signals including pupil diameter, heart rate, skin conductance, and subjective emotional experience in terms of valence and arousal. These signals were chosen because they are some of the most commonly studied in emotion research (Kreibig, 2010; Norman et al., 2016) and because their low cost and participant burden mean they are likely to continue to be used. The use of multiple measures allowed for estimating the degree of coherence between the different autonomic response indices. Given the mixed findings regarding the degree of coherence between different modalities (Kreibig, 2010; Norman et al., 2016), and suggestions of high variability between individuals (Quigley & Barrett, 2014; Wilson‐Mendenhall et al., 2015), no firm predictions could be made, but any observed coherence between modalities was expected to be small.

Second, and contrary to previous multivariate approaches which analyzed subjective and autonomic responses to emotion separately before analyzing the relationships between them (Christie & Friedman, 2004; Kim & Wedell, 2016; Sokolov & Boucsein, 2000; Stemmler, 2004), the organization of autonomic and subjective emotional responses was investigated within a common affective space. Contributions of autonomic and subjective responses to common dimensions within this autonomic‐subjective affective space would suggest coherence between physiological and subjective emotional responses, whereas predominantly orthogonal organization would indicate divergence. The use of multivariate analyses which include both physiological and subjective responses has the potential to identify relationships that would be difficult to detect using standard approaches or multiple independent tests. Specifically, the current approach can identify patterns of coherence between autonomic signals, and between autonomic signals and subjective experience, even when distinct subjective emotions are not accompanied by a distinct pattern of response in single autonomic modalities, when individuals do not display a measurable physiological response in some modalities for some emotional experiences, and when subjective emotional responses are accompanied by physiological changes in some modalities, but not others (all of which are factors characteristic of psychophysiological responses; e.g., Berntson et al., 1991; Kreibig, 2010). Motivated by previous meta‐analyses and reviews (Cacioppo et al., 2008; Hamm & Weike, 2005; Kreibig, 2010), it was expected that dimensions of the autonomic‐subjective affective space would encode subjective valence and arousal.

## 2 METHOD

All research procedures were approved by the institutional Ethics Committee and were in accordance with the revised 2013 Declaration of Helsinki. A total of 51 healthy participants (33 female; *M*_age_ = 24.88, *SD*_age_ = 7.15) recruited from a volunteer database took part in the study for either course credit or monetary compensation. The sample size was determined a priori based on simulations to detect small to medium effect sizes, in terms of linear relationships between autonomic and subjective experience in multilevel mixed models analyses – see *SM2. Details of power estimations*.

### 2.1 Emotion task

**FIGURE 1:**
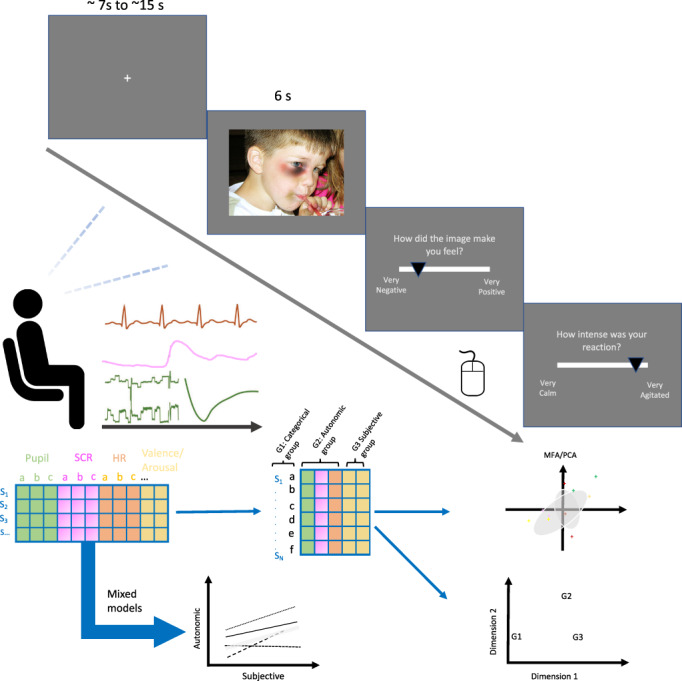
Schematics of experiment and analytical approach. Participants viewed emotional images for 6 s with an inter‐trial interval of 7–15 s, while their pupil size, eye movements, electrocardiogram, and skin conductance response were monitored. The lower panel illustrates the univariate multilevel regression approach and to the right the multivariate patterning approach.

Task structure is depicted in Figure 1. 56 images from the International Affective Picture System (IAPS, Lang et al., 2008) were used (See Supplementary materials: SM—Additional methods). The stimuli were selected to induce emotional states characterized by a wide range of valence (*M* = 5.07, *SD* = 1.92, Range = 1.46–8.19) and arousal (*M* = 4.66, *SD* = 1.19, Range = 2.63–8.21; nine‐point scales) according to norms provided in the stimulus set. Color versions of the images were used to preserve their vividness and naturalness, and image statistics (e.g., brightness, contrast) were computed and controlled for statistically in all pupil diameter analyses. Stimuli were displayed on a 1920 × 1200 pixels monitor, measuring approximately 25 (width) × 17 (height) degrees of visual angle at a 60 cm distance. Stimulus presentation was controlled in Psychopy (Peirce et al., 2019), running on an Intel Core i9 Windows 10 computer (32GB RAM), with a fixed refresh rate of 60 Hz.

Each trial started with a fixation cross of variable duration (ranging from 7 to 15 s), followed by the presentation of the stimulus image for 6 s, after which participants rated how the image made them feel using sliders—first for valence (“How did you feel watching the image?”); from “‐10 (Extremely negative)” to “+10 (Extremely positive)”, and then arousal (“How intense was your emotional reaction?”); from “‐10 (Very calm)” to “+10 (Very agitated”). Participants were asked to report how they “actually” felt viewing each stimulus, rather than how they thought they “should” feel.

### 2.2 Physiological recording and processing

#### 2.2.1 Eye movements and pupillometry

Eye movements and pupil changes were monitored using a GP3‐HD—Gazepoint (Cuve et al., 2022) eye‐tracker sampling at 150 Hz, calibrated prior to the task and after 25 trials. A chinrest was used to minimize head and body movements. Blinks and invalid samples (pupil diameters outside the range of 2–10 mm) were interpolated and smoothed linearly up to 150 ms. A subtractive baseline correction was applied using an interval corresponding to one second before and one second after the stimulus onset (Cuve et al., 2022).

#### 2.2.2 Skin conductance

Skin conductance data were recorded using the EDA100C module and TSR203 transducers on a BIOPAC MP160 system sampling at 2000 HZ. Data were collected via pre‐gelled disposable electrodes placed on the inside of the left foot and processed in Ledalab (Benedek & Kaernbach, 2010). Data were downsampled to 10 Hz, inspected for artifacts, and smoothed using the adaptive smoothing algorithm in Ledalab. Continuous Decomposition Analysis and Trough‐to‐Peak analyses (Benedek & Kaernbach, 2010) were implemented to identify stimulus‐related skin conductance responses (SCR) which were used for statistical analyses. Responses were defined as phasic signals detected between 1 and 6 s after stimulus onset.

#### 2.2.3 Heart rate

Heart rate (HR) was recorded via the ECG100C electrocardiogram amplifier with two disposable electrodes provided by the manufacturer (BIOPAC Inc), placed on each participant's right collarbone and lower left torso, and secured with surgical tape to prevent slippage. Heart rate was computed in AcqKnowledge software from BIOPAC, as an average heart rate following stimulus onset. A difference score was computed by subtracting mean heart rate after stimulus onset from average heart rate pre‐stimulus (during the fixation cross), and this difference score was used for statistical analyses.

### 2.3 Statistical analyses

#### 2.3.1 Univariate analyses

Linear mixed models were used to predict autonomic responses (pupil diameter, SCR, HR), separately, from reported subjective experience (valence and arousal) as fixed effects, while accounting for trial‐by‐trial and participant variability (see Sommerfeldt et al., 2019; DeBruine & Barr, 2021), by including participant and stimulus as random effects (see section 3.1) and to test pairwise relationships between the different signals (see section 3.2). Models were fitted using the R package lme4 (Bates et al., 2014), and approximate *p* values were derived using the lmerTest package (Kuznetsova et al., 2017). Full model details are provided in SM (Mixed model details).

#### 2.3.2 Multivariate dimensional analyses

A Multiple Factor Analysis (MFA) was used to explore the dimensional organization of autonomic and subjective emotional responses. MFA was chosen because it can incorporate subgroups of variables (e.g., autonomic variables and subjective ratings), without artefactually inflating the importance of a single subgroup, due to the number of variables it includes or differences in ranges (Abdi et al., 2013; Lê & Husson, 2008). MFA also allows the inclusion of categorical variables, such as stimulus, stimulus category (e.g., negative, positive, and neutral valence), and participants, as supplementary variables to assess their contribution to the construction of a common dimensional space, thus retaining (and identifying) the contributions of the different variable subgroups and individual variables to the global structure (Kassambara & Mundt, 2020; Lê & Husson, 2008). Conveniently, the results of the MFA are interpreted as one would interpret the results of a principal component analysis; however, because principal component analysis methods generally do not explicitly account for the hierarchical (multilevel) structure of the data, variants of the analyses detailed above were conducted by aggregating variable groups (autonomic and subjective) by participant and stimulus separately, as well as by participant and stimulus category, with the category representing averaged responses for negative, neutral, and positive stimuli (and low, medium and high arousal) defined using the normative stimulus ratings. These variants were used as sensitivity analyses to investigate the degree to which results changed due to the hierarchical structure in the data, and can also give insights **into** the most relevant factors that drive variance in the dimensional organization of autonomic and subjective variables (e.g., participants vs stimuli). These sensitivity analyses are also useful to understand whether some patterns may be limited to group‐level effects, as opposed to being replicable across participants.

The stability and significance of the MFA solution were assessed computationally, to investigate the robustness of the solution with respect to the underlying data. First, 95% confidence intervals (CI) for point estimates such as eigenvalues (to test which components should be retained), and loadings (quality of variable/dimension relationships), were computed using bootstrapping. Bootstrapping was performed by resampling with respect to the hierarchy in the original dataset, refitting the MFA on each simulated dataset, and storing the point estimates of interest (described above). This was performed 10,000 times and then the .025 and .975 quantiles were computed to derive 95% CIs around the estimates of interest, based on the bootstrapped distribution.

Second, non‐parametric statistical testing was conducted using resampling approaches that consisted of reshuffling the quantitative variables separately (individually) while respecting the hierarchical structure (e.g., reshuffling within‐participant/stimulus category), to simulate “null” datasets with no real relational structure in the data (i.e., any relationship between autonomic and subjective variables occurs by chance). An MFA was then refitted to each simulated dataset. This process was repeated 10,000 times, storing the point estimates at each iteration (eigenvalues, loadings), to create a null distribution for the MFA estimates. This was used to compute the probability of obtaining MFA solutions and estimates that were directionally (one‐tailed) more extreme than that of the observed data, under the null distribution at *p* < .05. The application (and suitability of bootstrapping and resampling approaches) of principal component methods have been reviewed and discussed extensively elsewhere (Abdi et al., 2013; Abdi & Williams, 2010). Finally, to confirm the informativeness of the MFA results, we used the dimensions for clustering: to find groupings (suggestive of distinct states) in a data‐driven fashion, and classification (based on a random forest approach) to distinguish a priori‐defined state groupings (Husson et al., 2011; James et al., 2013) based on valence (see SM5. Classification and clustering analysis details).

## 3 RESULTS

Results are organized into three subsections, namely (1) Coherence between individual autonomic signals and subjective experience. (2) Coherence between different autonomic signals. (3) The autonomic‐subjective affective space: multivariate analysis of autonomic and subjective emotion responses in a common space.

### 3.1 Coherence between individual autonomic signals and subjective experience

#### 3.1.1 Pupil diameter and subjective emotion

**FIGURE 2:**
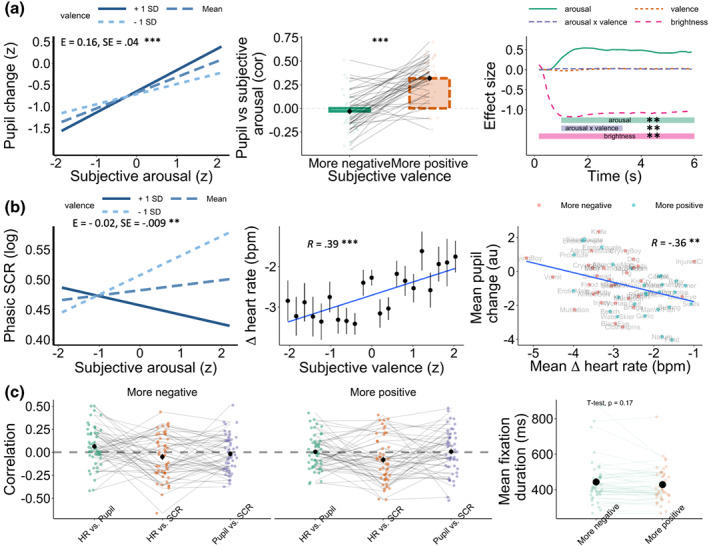
Summary of autonomic and subjective coherence regression analyses. (a) Subjective arousal and pupil size showed higher coherence for positive valence trials (+ 1SD). (b) Illustration of the arousal and valence interaction showing higher coherence between subjective arousal and skin conductance response (SCR) for positively compared to negatively valenced states. Heart rate tracked subjective valence responses (middle) where error bars represent aggregated responses for each stimulus across participants. Right—Illustration of the only significant aggregate relationship between physiological signals (note that data is aggregated by stimulus, across participants). (c) Illustration of average cross‐correlations between autonomic signals, revealing low coherence in autonomic responses (i.e., weak associations between autonomic responses). Each dot represents a participant and data is faceted by valence (median split for visualization). Data from the same participants are connected across conditions by lines. *p < .05, **p < .01, ***p < .001.

Subjective arousal predicted pupil diameter on a trial‐by‐trial basis as expected (Estimate = .36, *SE* = .05, *t* = 7.48, *p* < .001), even after accounting for stimulus valence and brightness. The main effect of valence was not significant (Estimate = .03, *SE* = .04, *t* = 0.68, *p* = .50). There was a significant valence by arousal interaction (Estimate = .16, *SE* = .04, *t* = 4.14, *p* < .001), indicating an asymmetric relationship between subjective arousal and pupil diameter, with higher concordance between subjective arousal and pupil size when participants reported feeling more positive (see Figure 2a). These results could not be explained by eye‐movement fixation patterns, which were not predictive of subjective ratings (see Figure 2c). As expected, there was a strong effect of brightness (Estimate = −.96, *SE* = 0.05, *t* = −17.76, *p* < .001), with brighter stimuli‐inducing greater pupil constriction (but no relationship between brightness and subjective valence or arousal).

In summary, these results suggest that the relationship between subjective arousal and changes in pupil diameter is somewhat dependent on subjective valence. Furthermore, while the visual representation of the arousal by valence interaction shows higher coherence between subjective arousal and pupil diameter for positive valence experiences (Figure 2a), a subset of participants showed the opposite effect, with subjective arousal and pupil diameter showing more coherence for negative subjective experiences. This suggests the presence of individual differences in the relationship between physiological reactivity and subjective arousal, which are typically overlooked in previous studies relying only on analyses of aggregated (group‐level) patterns of physiological activity and subjective experiences (see also Supplementary materials—Temporal analysis of pupil diameter).

#### 3.1.2 Skin conductance and subjective emotion

Both subjective arousal (Estimate = 0.02, *SE* = 0.01, *t* = 2.60, *p* < .01) and valence (Estimate = −0.03, *SE* = 0.01, *t* = −2.51, *p* < .01) were significant predictors of SCR individually, but not when included within the same model to predict event‐related SCR. This suggests that the subjective arousal and valence effects on skin conductance are likely due to common variance in these ratings. As with the pupil diameter results, there was an interaction between subjective arousal and valence, suggesting that the relationship between subjective arousal and SCR is modulated by subjective valence (Estimate = −0.02, *SE* = −.009, *t* = −2.77, *p* < .01). In contrast to the pupil diameter data, however, subjective arousal and SCR responses were more coherent during negative rather than positive states (see Figure 2b).

#### 3.1.3 Heart rate and subjective emotion coherence

Change in heart rate was predicted by subjective arousal (Estimate = −0.28, *SE* = 0.09, *t* = −3.04, *p* < .01) and valence (Estimate = 0.29, *SE* = 0.08, *t* = 3.28, *p* < .01), such that heart rate decelerated when participants reported higher arousal and more negative emotion. However, when tested in the same model, only valence was significant (Estimate = 0.22, *SE* = 0.001, *t* = 1.94, *p* < .05), such that more negative stimuli induced a stronger heart rate deceleration, whereas more positive stimuli induced heart rate acceleration (see Figure 2b).

### 3.2 Coherence between autonomic responses

Analyses investigated the degree to which the different autonomic signals could be predicted from each other, to provide a test of coherence within autonomic responses following emotion induction (Figure 2c). While both pupil diameter and skin conductance tracked subjective arousal in the previous analyses, they could not be predicted from one another on a trial‐by‐trial basis. Instead, they could only be predicted by changes in HR. Thus, SCR was significantly predicted by HR (Estimate = −0.005, *SE* = 0.002, *p* < .001), but not pupil diameter (Estimate = −0.006, *SE* = 0.005, *p* = 0.224). Trial‐by‐trial fluctuations in pupil diameter were predicted by changes in HR (Estimate = 0.03, *SE* = 0.007, *p* < .001), but not by SCR (Estimate = −0.04, *SE* = 0.03, *p* = .240). It is worth noting, however, that even the significant effects are modest, with large amounts of between‐subject variance (see Figure 2c), questioning the interchangeable use of different autonomic modalities to measure emotional arousal.

### 3.3 The autonomic‐subjective affective space: Multivariate analysis of autonomic and subjective emotion responses in a common space

**FIGURE 3:**
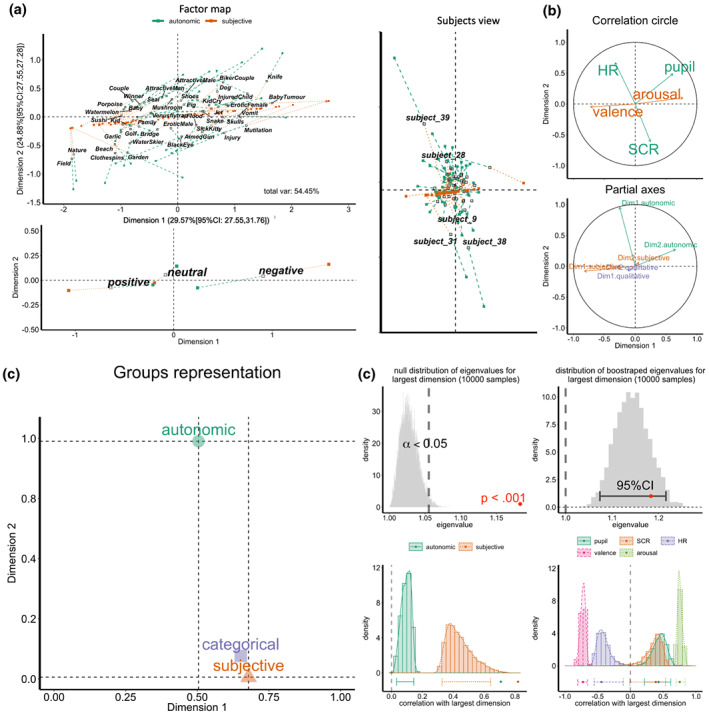
Multivariate dimensional analyses of autonomic and subjective affective responses. (a) Factor maps depicting the distribution of stimuli, participants, and stimuli categories (both included in the class “categorical variables” in c) on the identified dimensions. Unfilled markers represent the mean (barycentre) of autonomic and subjective response loadings, while colored dashed lines and filled boxes from the unfilled markers represent autonomic (green) and subjective (red) loadings. The extent to which these autonomic and subjective markers diverge in the construction of the dimensions indicates the degree of coherence (longer lines in opposite directions = less coherence). (b) Correlation circle shows the quality of variable‐dimension relationships (closer to perimeter = good representation of variable responses by dimensions). Partial axes show the relationship between global and partial (autonomic and subjective combined vs separate, respectively) PCAs. (c) Representation of the overall contribution of variable groups (autonomic, subjective, and categorical). Of most interest is the fact that both autonomic and subjective variables contribute to dimension 1, while dimension 2 has almost no contribution from subjective responses despite a substantial contribution from autonomic variables. (d) Summary of bootstrapping and resampling analyses (10,000 iterations) to assess the stability of the autonomic‐subjective affective space.

Unlike the previous analysis, the MFA allows investigation of the relationships between all autonomic and subjective variables and how they organize into underlying dimensions (see Method). The MFA consistently suggested two significant components that effectively represented the covariance structure in autonomic and subjective responses to emotion induction. The main solution explained a total of 53.51% of the variance, with the stability of the solution estimated by resampling and bootstrapping simulations (*p* < .001; see Figure 3). Both subjective and autonomic variable covariance contributed significantly to dimension 1 (29.38% [95% bootCI: 27.55, 31.76]). In contrast, dimension 2 (25.13% [95% bootCI: 22.97; 27.28]) included significant autonomic covariance almost exclusively.

Figure 3 (Panel a) shows the factor maps for the first two dimensions showing the mean (barycentre) distribution of stimuli (unfilled) loadings for autonomic (green) and subjective (red) variables. There are two noteworthy patterns, the first is that dimension 1 maps loosely onto subjective valence (and to a lesser extent subjective arousal—both inferences derived from the semantic content of the stimuli and participants' ratings on the same dimensions), with dimension 2 being less readily interpretable. The second pattern is illustrated best by the projection of autonomic and subjective variable group partial views (green and red dashed lines and points) projecting away from the barycentres (central unfilled markers). The direction of these projections away from the central markers suggest that the contribution of autonomic and subjective groups to the construction of the dimensions is somewhat divergent.

The same pattern can be seen more clearly when projecting valence categories based on the IAPS normative data (Figure 3a, bottom left) or when projecting participants to the factor Map (Figure 3a, right).

The correlation circle (Figure 3b) depicts the quality of variable‐dimension relationships, with variables closer to the circle perimeter contributing more strongly to the creation of the respective dimensions. It highlights two additional noteworthy patterns, first, that both subjective valence (cor = −0.725) and arousal (cor = 0.774) correlate with Dimension 1. This reflects the finding that the subjective intensity of emotional response to these stimuli is negatively related to valence (negative stimuli tended to generate a stronger subjective sense of arousal). This relationship was particularly well captured by dimension 1 as indicated by the orientation of both variables along the x‐axis and their distance from the origin (close to the perimeter). All three autonomic responses were also correlated with dimension 1. The second pattern that is evident in the correlation circle is the lack of correlation between subjective responses and dimension 2, despite correlations between all three autonomic responses and dimension 2.

The results thus far demonstrate both coherence and divergence between autonomic and subjective emotional responses. Specifically, the significant covariance between autonomic and subjective responses that are captured by dimension 1 indicates coherence. However, the significant amount of shared variance in autonomic responses captured by dimension 2 is not reflected in subjective responses and thus indicates divergence. These interpretations are reinforced by variable group contributions to the dimensions (Figure 3c,d), with results suggesting that subjective variables contribute far more strongly to dimension 1 (cos2 = 0.44, contribution = 57.38%) than 2 (cos2 = 0.01, contribution = 0.72%), whereas autonomic responses contribute strongly to Dimension 2 (cos2 = 0.37, contribution = 99.28%) and moderately to dimension 1 (cos2 = .09, contribution = 42.61%).

Finally, the partial axes (Figure 3b, bottom), relate the separate PCAs on each variable group (subjective and autonomic) to the global PCA. This plot shows that subjective response organization and their contribution to the global PCA space are similar when only subjective variables are analyzed. For the autonomic group, however, the first dimension in the separate autonomic PCA contributes to the second dimension in the global PCA, whereas the second dimension of the autonomic PCA contributes primarily to the construction of the first global dimension. This suggests that the main driver of divergence in the autonomic‐subjective space is related to the organization of autonomic responses. Specifically, autonomic responses appear to have two significant covariance profiles, one varying along a subjective valence‐arousal dimension (derived from stimulus semantics), and one that does not but shows a strong covariance between SCR and heartrate and which explains the majority of the variance of autonomic responses.

Importantly, the overall pattern remained consistent when the MFA analyses were conducted on aggregate data, by participant, and by stimuli with the latter explaining a greater amount of variance and reducing the divergence between subjective and autonomic responses (see SM—Additional MFA analysis). This suggests that individual differences in autonomic and/or subjective responses might be one of the factors underlying reduced coherence between these responses at the group level.

#### 3.3.1 Validation of autonomic‐subjective affective space

**FIGURE 4:**
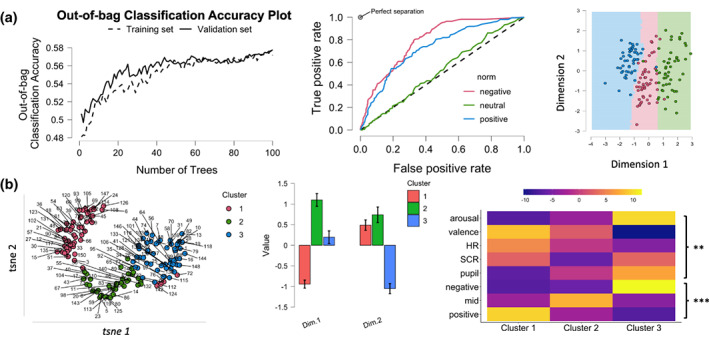
Validation of the autonomic‐subjective affective space. (a) classification results based on random forest. (b) Data‐driven clustering results based on *kmeans*. The decision boundary matrix for classification (top right) and the plot for clustering (lower left) are based on aggregated data by the stimulus for illustration purposes only, to reduce overplotting. Heatmap (bottom right) shows the contribution of individual variables to clustering and a significant correspondence between classification and clustering results. **p < .01, *** < .001.

One way to test the validity of the autonomic‐subjective affective space results is to explore whether this low‐dimensional organization of autonomic and subjective response can be used for classification (a priori, theory‐driven) or clustering of emotional states (a‐posteriori, data‐driven). For a priori theory‐driven classification, a random forest classifier was trained to predict the stimulus class (negative, neutral, positive valence, based on IAPS normative data) from the two dimensions of the autonomic‐affective space—(see SM Classification and Clustering analysis details). This provided an accuracy between 50% (test data) and 60% (out‐of‐bag, note that chance for classifying three classes is 33%), and good receiver observer characteristics (see Figure 4), with area under the curve (AUC = the degree or measure of separability) values ranging from .5 to .8, with a significant classification matrix (*X* ^2^(4) = 271.304, *p* < .001). Crucially, dimension 1 (indicating coherence between subjective and autonomic response) had higher variable importance (Mean decrease in accuracy = .2) than dimension 2 (dominated by autonomic structure that did not reflect subjective experience—Mean Decrease in accuracy = −.01).

Data‐driven clustering conducted on the autonomic‐subjective affective space dimensions using k‐means provided an optimal solution with three clusters (see SM—Classification and Clustering analysis details). This solution explained 65% of the variance and had a silhouette score of .42, showing imperfect but significant separation between clusters (0 = no clusters, 1 = perfect separation). A chi‐square test confirmed that the identified clusters were consistent with the three classes used for a priori, theory‐driven classification (negative, neutral, and positive valence classes based on IAPS norm data) at an above chance level (*X* ^2^(4) = 138.56, *p* < .001)—Figure 4b.

In summary, we find a low‐dimensional organization of the autonomic‐subjective affective space that indicates both coherence and divergence in autonomic and subjective emotional responses. Specifically, a moderate degree of coherence was captured by the first dimension, whereas the second dimension suggested the presence of robust covariance between autonomic signals that did not reflect subjective affective experience.

## 4 DISCUSSION

The current study investigated the relationship between autonomic responses to emotion, and between those autonomic responses and subjective responses to emotion. Two analytical approaches were used, first, a univariate approach that accounted for trial‐by‐trial and individual variability in the relationship between autonomic and subjective emotional responses, and second, a multivariate dimensional approach which enabled investigation of the organization of autonomic and subjective emotional responses in a common affective space.

Univariate analyses indicated only a very limited degree of coherence between autonomic signals; although pupil diameter and skin conductance could be predicted from changes in heart rate, effect sizes were small, and pupil diameter could not be predicted reliably from skin conductance. The coherence between autonomic and subjective emotional responses was no higher. Pupil diameter predicted subjective arousal in a valence‐specific manner—coherence was higher for positive subjective affective states (although there were substantial individual differences in this pattern, with some participants showing increased coherence for negative states). Skin conductance also predicted subjective arousal in a valence‐specific manner, although in this case coherence was higher for negative affective states. In contrast, changes in heart rate predicted subjective valence but not arousal. As in the between autonomic signals analyses, relationships between subjective and autonomic emotional responses were modest and subject to substantial individual differences.

The multivariate dimensional analysis indicated both coherence and divergence between autonomic and subjective emotional responses. The first dimension had contributions from both autonomic and subjective responses and therefore indicated a degree of coherence, however, autonomic responses had an additional covariance pattern that contributed almost exclusively to the second dimension. This latter finding indicates that there is substantial structure in autonomic responses which does not reflect subjective emotional experience (at least as captured by valence and arousal).

With respect to the results of the univariate analyses, the observed moderation of the association between autonomic signals and subjective arousal by valence is incompatible with previous suggestions that such signals track arousal regardless of valence (Bradley et al., 2008; Collet et al, 1997). However, moderation of the autonomic‐subjective arousal relationship by valence has been observed previously for other autonomic signals such as blood pressure and several other cardiovascular metrics (Cacioppo et al., 2008; Kreibig, 2010; Siegel et al., 2018), and was suggested by both early and recent theories in the physiology of emotion literature (Brown et al., 1951; Taylor, 1991; Wendt et al., 2022).

A key feature of the moderation of the autonomic‐subjective arousal relationship by valence is the extent to which it was subject to individual differences, with some participants exhibiting the opposite effect to that observed at the group level or no moderation at all. Indeed, substantial individual differences were observed in all demonstrations of a degree of coherence between autonomic signals and between autonomic signals and subjective responses. These findings, therefore, suggest that the relationship between physiological responses and subjective emotional experience might be idiosyncratic—at least to a degree—as proposed by some modern theories of emotion (Barrett, 2006; Picard et al., 2001; Wilson‐Mendenhal et al., 2015). This in turn may drive much of the variability in autonomic responses to subjective emotion reported in several reviews of the existing literature (Kreibig, 2010; Norman et al., 2016; Siegel et al., 2018). Future research should attempt to identify and account for individual differences explaining this variability (e.g., interoceptive sensibility [Barrett et al., 2004], Critchley & Garfinkel, 2017) and subclinical traits like alexithymia (Brown et al., 2020; Gaigg et al., 2018; Hickman et al., 2021; Van Doren et al., 2021). It also seems pertinent to move toward the adoption of analytical approaches capable of formally assessing the prevalence of coherence within and between autonomic and subjective emotional responses. Bayesian population prevalence analysis could be informative in this regard, as it allows inference of the expected prevalence of an effect in a certain population from the proportion of participants showing that effect (e.g., coherence), and/or the size and direction of the effect (Ince et al., 2021).

With respect to the results of the multivariate dimensional analysis, it was argued that studying the covariances between autonomic and subjective affective responses in a common space may reveal relationships between autonomic and subjective responses that are not always apparent when using either univariate approaches or multivariate approaches which look for patterns in autonomic and subjective responses separately. Using this approach, we found a two‐dimensional autonomic‐subjective affective space that suggests the existence of both coherence and divergence in autonomic and subjective affective responses. The first dimension mapped reasonably well to subjective valence and arousal (in a non‐orthogonal manner), with significant covariance with autonomic signals suggesting a degree of coherence. These results are consistent with dimensional views of emotion which propose that emotional events can be represented in a lower‐dimensional space that organizes the subjective and autonomic components of emotional experience (Barrett, 2006).

However, the second dimension was dominated by autonomic patterns that did not covary with subjective affective response, therefore suggesting a degree of divergence. Interestingly, the autonomic covariance patterns in the second dimension resemble the well‐known orienting response, which reflects a neurophysiological reaction to novel or significant stimuli that is thought to optimize perception and behavioral response (Graham & Clifton, 1966; Sokolov, 1963; Wendt et al., 2022). Thus, these data are consistent with the idea that the emotional events consist of both emotion‐specific autonomic responses (that can differentiate subjective experience, at least to a degree), and a non‐specific orienting response (Stemmler, 2004). Furthermore, the orienting response appears to be a more dominant feature of autonomic response to emotion induction than previously thought and may explain the generally small degree of coherence between autonomic and subjective responses observed in the literature (Kreibig, 2010; Norman et al., 2016; Siegel et al., 2018, although see Levenson, 2014).

The utility of the multivariate approach adopted here is evident when considering suggestions that a many‐to‐one (or one‐to‐many) relationship may exist between subjective affective states and autonomic responses—many different patterns of autonomic activity may be associated with the same subjective emotional experience, **or** one autonomic pattern to many different subjective experiences of emotion (Cacioppo et al., 2008, Levenson, 2014). Such “degeneracy” is a thought to provide increased robustness in many biological systems, including neuroanatomical, neuronal, and autonomic systems (Edelman, 2001; Friston & Price, 2003). Modern theories of emotion (e.g., Barret, 2017; Quigley & Barrett, 2014), and recent experimental evidence, supports the existence of degeneracy in affective brain networks (Doyle et al., 2022). Multivariate dimensional approaches are better suited to investigate degenerate systems, as they do not require assumptions regarding the independence or otherwise of variables, do not require dimensions or categories of emotion to be specified in advance, and allow for differential patterning of autonomic and subjective responses based on patterns of covariance. For these reasons, we argue that more experimental studies should adopt multimodal measurement of autonomic and subjective responses, and multivariate analytical approaches, to fully map the autonomic‐subjective affective space. While multivariate analytical approaches might suggest an atheoretical approach to the study of emotion, this need not be the case. It is possible to integrate and test theoretical predictions and benefit from data‐driven discovery at the same time. Using these approaches future research could directly address longstanding debates regarding categorical vs. dimensional organization of emotional responses by evaluating whether there are distinct and homogenous (or even distinct but overlapping) clusters in the affective space that reflect specific emotion categories and contexts, and identify the most diagnostic autonomic and behavioral features of subjective affective experience.

Increasing research using multivariate and multimodal approaches to study emotion will also inform as to the suitability of the various data‐driven approaches to characterize the autonomic‐subjective affective space. For example, there are some limitations that are not easily addressed by standard multivariate dimensional approaches, including the fact that most rely on aggregated data to find dimensions or clusters, and are not tailored for non‐independence in data which typically is present in repeated‐measures experiments (including multiple data points within participants). While hierarchical (multilevel) dimensionality reduction approaches do exist, they constitute an area of active research and development (Bouhmala et al., 2016; Di et al., 2014) and have not been tested using psychophysiological data. The ideal approach should allow for exploring dimensions and or clusters within and across individuals such that both individual differences and commonalities in autonomic and subjective emotional responses are captured. Recent advances in data‐driven methods from unsupervised clustering to deep learning approaches appear particularly promising in this regard for enabling the identification of consistent and inconsistent patterns in emotion responses (Doyle et al., 2022; Picard et al., 2001).

Two issues that need to be considered in the future both have the potential to impact the degree of coherence seen between autonomic and subjective affective responses. The first is that of timescales—most research has averaged responses over intervals that are typically determined by features of stimulus presentation, yet both autonomic and subjective responses are likely to evolve over time, at different rates, and with different response profiles. Some have argued that coherence between emotional responses may be much higher when temporal coherence approaches are used (Levenson, 2014), however, it remains unclear what the appropriate timescales are, particularly given that not all physiological signals have a clear response profile (e.g., pupil variables, facial expressions, movement). The second issue that may impact coherence is that of emotion regulation, and whether, how, and how quickly, it modulates emotional responses. Emotion regulation may not affect all autonomic modalities equally and may act to reduce coherence between autonomic responses and between autonomic responses and subjective responses (Dan‐Glauser & Gross, 2011; Levenson, 2014; Stemmler, 2004). A lack of coherence, therefore, does not necessarily imply no underlying relationship between autonomic states and subjective affective experience.

Finally, the measure of arousal and how it relates to valence warrants further consideration. Differences between our results and previous studies may reflect differences in the measurement of arousal intensity, operationalized on a continuum from low arousal to high arousal which reflecting a change in state from calm to agitated/excited. Variation in the measurement of perceived arousal may lead to different relationships between arousal and autonomic responses, possibly in a valence‐dependent fashion, which varies across individuals (see Kuppens et al., 2016).

Despite the hurdles to be overcome, refinement of the autonomic‐subjective affective space may prove useful in testing theories of emotion and also for understanding atypical emotional responses. For example, theories of autism (Garfinkel et al., 2016; Hadjikhani et al., 2018; Quattrocki & Friston, 2014) and alexithymia (Brewer et al., 2016; Nemiah, 1976) often claim that the emotional difficulties in these conditions reflect atypical mobilization of, or reduced coherence between, autonomic and subjective responses to affective stimulation. Despite these theories, it has proved difficult to identify a single “clinical” profile based on autonomic markers, and the literature has suffered from many of the problems encountered in the study of typical individuals (Arora et al., 2021; Cuve et al., 2018; Panayiotou et al., 2018). It would be interesting to determine whether members of atypical groups such as autistic or alexithymic individuals can be localized in common—and distinct—areas of a refined autonomic‐subjective affective space.

## AUTHOR CONTRIBUTIONS

**Hélio Clemente José Cuve:** Conceptualization; data curation; formal analysis; funding acquisition; investigation; methodology; project administration; resources; software; visualization; writing – original draft; writing – review and editing. **Joseph Harper:** Data curation. **Caroline Catmur:** Supervision; writing – review and editing. **Geoffrey Bird:** Conceptualization; funding acquisition; resources; supervision; writing – review and editing.

## FUNDING INFORMATION

HCC was supported by a Medical Sciences Division Graduate Studentship awarded by the Clarendon Fund and the Kendrew Fund (St John's College, University of Oxford). GB was supported by the Baily Thomas Charitable Trust. This work was partly funded by the Economic and Social Research Council (Grant: ES/R007527/1).

## CONFLICT OF INTEREST STATEMENT

None.

## Supporting information


**Data S1:** Supporting information

## Data Availability

Data and code for this article are available at: https://github.com/hcuve/ASAS.
