# Supplementary material for: Coherence and divergence in autonomic‐subjective affective space
Source: Psychophysiology. 2023 Feb 5;60(6):e14262. doi: 10.1111/psyp.14262 (PMC10909527; doi:10.1111/psyp.14262)
Supplement: Supplementary file 1 — Data S1: Supporting information [file PSYP-60-e14262-s001.pdf]

Supplemental materials

SM1: Additional Methods
List of IAPS stimuli used in the current study (P. Lang & Bradley, 2007; P. J. Lang, Bradley, & Cuthbert, 1997).
Note that IAPS stimuli cannot be shared publicly, below is the list of the stimuli used in the current study.

1019.jpg; 1302.jpg; 1350.jpg; 1440.jpg; 1920.jpg; 2035.jpg; 2071.jpg; 2156.jpg; 2301.jpg; 2345.1.jpg; 2374.jpg; 2392.jpg; 2400.jpg; 2457.jpg; 2550.jpg; 2770.jpg; 2900.1.jpg; 3010.jpg; 3069.jpg; 3103.jpg; 3130.jpg; 3170.jpg; 3185.jpg; 3301.jpg; 4230.jpg; 4311.jpg; 4530.jpg; 4573.jpg; 4574.jpg; 4631.jpg; 4800.jpg; 5040.jpg; 5199.jpg; 5530.jpg; 5665.jpg; 5725.jpg; 5760.jpg; 5836.jpg; 6250.jpg; 6555.jpg; 7026.jpg; 7038.jpg; 7052.jpg; 7325.jpg; 7354.jpg; 7477.jpg; 8200.jpg; 8312.jpg; 8330.jpg; 9320.jpg; 9440.jpg; 9472.jpg; 9561.jpg; 9622.jpg
9800.jpg; 9926.jpg

SM2. Details of power estimations

Estimates of the degree of coherence between subjective affective experience and autonomic response in the literature have been heterogeneous, ranging from small to moderately large effect sizes (Kreibig, 2010; Norman et al., 2016, Siegel et al., 2018). Based on data from our previous studies (Cuve et al., 2022, Gaigg et al., 2018), we assumed an average effect size of approximately .4 (Cohen's d, upper bound for a small effect). For a target power of 80% and an alpha of <.05, it results in approximately 50 participants (see power contours A & B). This means that for a population where a true effect is zero (C left), we would detect the target effect about 80% of the time (C right density).

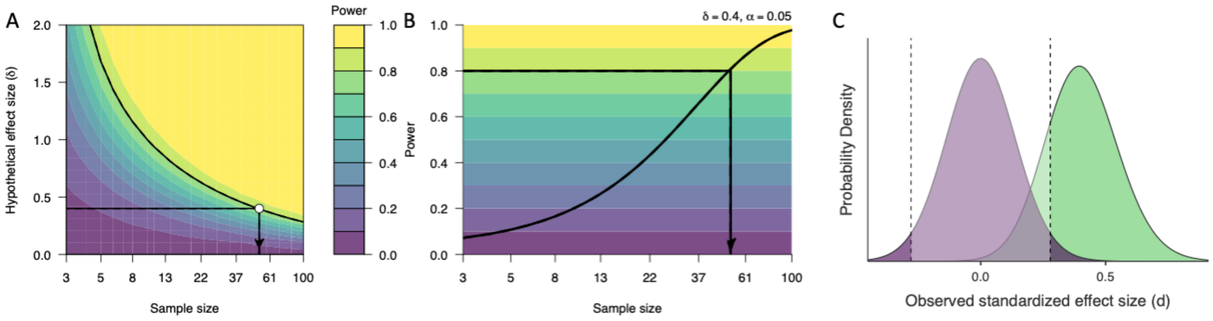

Figure S1. Illustration of effect sizes and power estimation as a function of the sample (effect size of .4, alpha .05, 80% power).

Note however that the final sample reflects a practical choice, as, in practice, power will vary with each predictor and a range of model optimisation decisions (e.g. estimating slopes or not), the optimality of which cannot be determined apriori and often needs to be driven by the data at hand (see Bates, Kliegl, Vasishth & Baayen, 2015). However, given that most effect sizes from previous studies are derived from aggregated (averaging) approaches, which are less powerful than the multilevel approach used here, we were confident that our sample estimation should provide enough power for the current analysis.

SM2: Temporal analysis of pupil diameter

There was a possibility that the relationship between arousal and pupil diameter may evolve over time, as suggested by recent studies showing distinct temporal components of the pupil response to emotional states (Kinner et al., 2017). To investigate this, linear mixed models were fitted to predict pupil size from subjective ratings of arousal and valence, their interaction, and image statistics (brightness, contrast) at different time points (n = 60, each bin being an average of 10 time points = 100 ms). Given the rapid pupillary light reflex (PLR) expected with visual stimuli, significant relationships between subjective arousal, valence and pupil are only plausible after the PLR (~ 1 second).

Significant time clusters were therefore defined as adjacent time bins where the corrected p-value remained < .05 after the PLR. The results of these analyses were consistent with the previous aggregated analyses (aggregated across time, not across participants or trials – see Figure 2-B). Specifically, there was a large effect of brightness (Mean effect $size_{0.3\text{-}6\ s} = -1.02$, $p < .001$). There was also a significant effect of subjective arousal, with more subjectively arousing stimuli associated with larger pupil dilation (Mean effect $size_{1\text{-}6s} = 0.45$, $p < .05$, corrected). The interaction between subjective arousal and valence had a small but significant

effect (Mean effect size $_{1\text{-}3s}$ = 0.03, $p < .05$, corrected), suggesting that early changes in pupil diameter following the light reflex are differently modulated by arousal and valence. As in the aggregated analysis, there was no significant main effect of valence (Mean effect size = 0.02, $p > .05$, corrected) suggesting that pupil diameter is primarily associated with arousal.

SM3. Mixed model details

SM3.1 Pupil mixed model results

		DV: Pupil	
Predictors	*Estimates*	*CI*	*p*
(Intercept)	-0.67	-0.93 – -0.42	**<0.001**
valence	0.03	-0.06 – 0.13	0.497
arousal	0.36	0.27 – 0.46	**<0.001**
Luminance	-0.97	-1.07 – -0.86	**<0.001**
valence * arousal	0.13	0.07 – 0.20	**<0.001**
**Random Effects**			
σ^2	2.60		
τ_{00} participant	0.86		
τ_{11} participant.Mean_gray	0.11		
τ_{11} participant.valence	0.01		
ρ_{01} participant	0.49		
ICC	0.27		
N_{ssid}	55		
Observations	2804		
Marginal R^2 / Conditional R^2	0.244 / 0.451		

All predictors are mean centred and standardised

SM3.2. SCR mixed model results

	DV: SCR		
Predictors	*Estimates*	*CI*	*p*
(Intercept)	0.48	0.37 – 0.59	**<0.001**
arousal	0.01	-0.02 – 0.04	0.540
valence	-0.03	-0.05 – 0.00	0.082
arousal * valence	-0.02	-0.04 – -0.01	**0.006**
**Random Effects**			
σ^2	0.21		
$\tau_{00\ stimIAPS}$	0.01		
$\tau_{00\ ssid}$	0.14		
ICC	0.42		
N_{ssid}	51		
$N_{stimIAPS}$	56		
Observations	2687		
Marginal R^2 / Conditional R^2	0.005 / 0.422		

SCR was converted into a logarithmic scale to correct for non-normality; therefore, the estimates reflect logarithmic distances. All predictors are mean-centred and standardised.

SM3.3 Heart rate

	DV: Heart rate change		
Predictors	*Estimates*	*CI*	*p*
(Intercept)	-2.73	-3.12 – -2.33	**<0.001**
arousal	-0.16	-0.37 – 0.06	0.149
valence	0.22	0.00 – 0.43	**0.046**
arousal * valence	-0.05	-0.18 – 0.09	0.486
**Random Effects**			
σ^2	15.09		
$\tau_{00\ stimIAPS}$	0.21		
$\tau_{00\ participant}$	1.48		
ICC	0.10		
$N_{participant}$	50		
$N_{stimIAPS}$	58		
Observations	2749		
Marginal R^2 / Conditional R^2	0.007 / 0.107		

All predictors are mean-centred and standardised.

SM - Autonomic-Subjective Affective space

SM4: Additional MFA results

MFA sensitivity analysis conducted on aggregated data (by participant * stimulus class: positive, negative, neutral; by stimulus id and by participant id) produced consistent results with those reported in the paper - see Figure SM4.1.

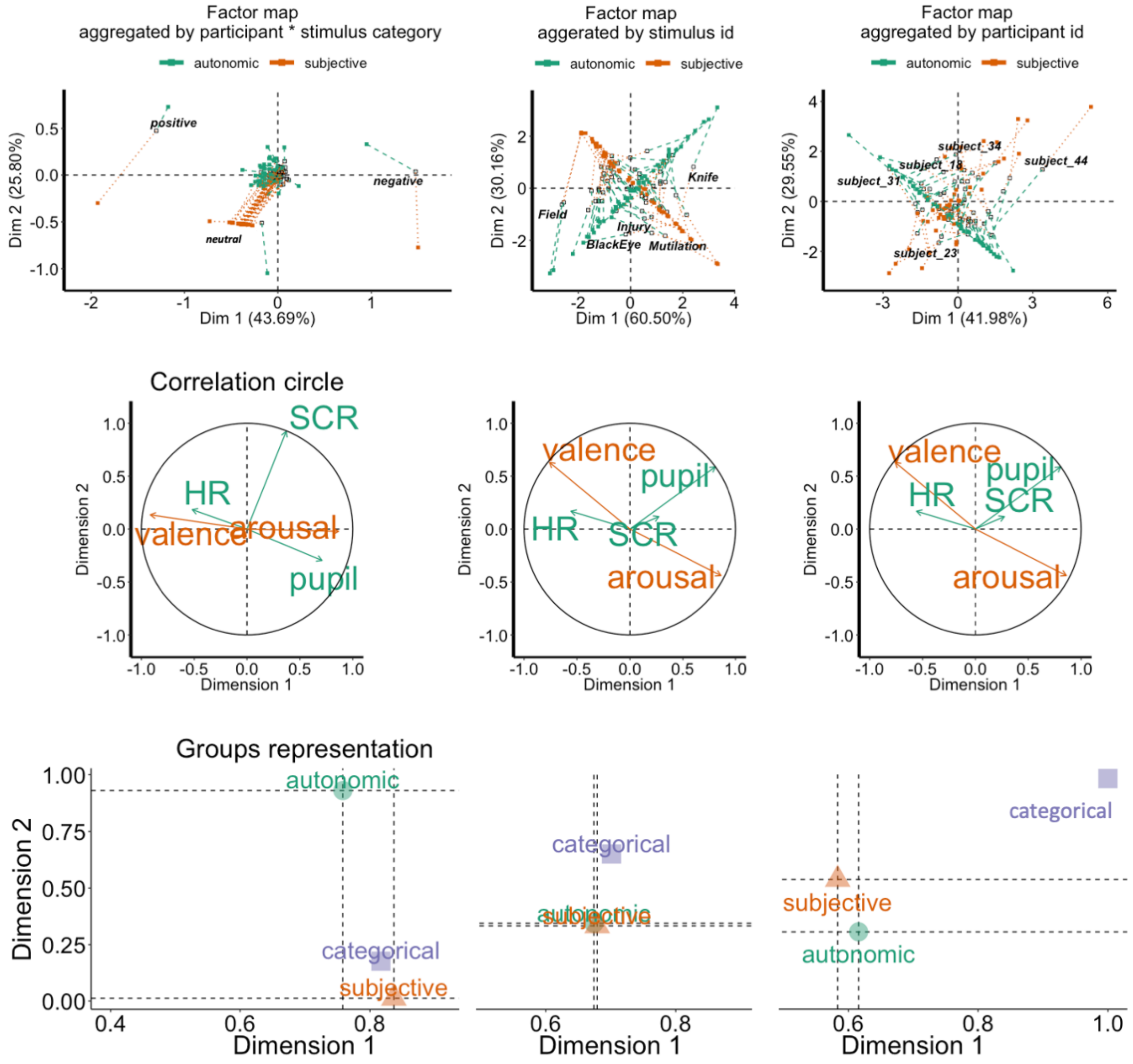

Figure SM4.1 Additional MFA results from sensitivity analysis.

Left column reflects results for data averaged by participant * stimulus category (averaged across stimulus within each category: negative, positive, neutral). Middle column shows results for data aggregated by stimulus (averaged across participants). Right column shows results for data aggregated by participant (average across stimuli id and category).

SM - Autonomic-Subjective Affective space

Factor maps show the most influential points in the autonomic-subjective affective space dimensions. Unfilled markers represent the mean (barycentre) of autonomic and subjective response loadings, while coloured dashed lines and filled boxes from the unfilled markers represent autonomic (green) and subjective (red) loadings. The extent to which these autonomic and subjective markers diverge in the construction of the dimensions indicates the degree of coherence (longer lines in opposite directions = less coherence). Correlation circle (middle row) shows the contribution of individual autonomic and subjective measures to the factor map (direction of arrow) and the quality of their representation by the dimensions (closer to perimeter = good representation). Third row is the representation of overall contribution of variable groups (autonomic, subjective and categorical) to the autonomic-subjective affective space dimensions.

Optimal solutions had two dimensions that captured covariance patterns in autonomic and subjective response to emotion induction. However, these results suggest that reduced coherence between subjective and autonomic responses is likely related to individual differences between participants, because analyses that aggregated responses by participant explained less variance than those that aggregated responses by stimulus (averaged across participants) – see Figure SM4.1.

SM5. Classification and Clustering analysis details

A supervised Random forest classifier was used to classify stimuli category (labels derived from the IAPS validation, Lang & Bradley, 2007) from the autonomic-subjective affective space dimensions. The classifier training set consisted of 60% of data points which was validated in held-out validation dataset making up 20% of the sample data, and test set of 20% of the data, all randomly partitioned. The number of decision trees was optimised according to out-of-bag error validation, from a maximum of 100 trees. Random forest analysis were conducted using functions in the R package randomForest (Liaw & Wiener, 2002).

Unsupervised data driven clustering was conducted using k-means. The optimal number of clusters (k) was tested by running the k-mean algorithm with $k = 2$ to $k = 10$ and computing the within-cluster sum of squares (WCSS) and plotting the results using the elbow method (the selected model being the model with the lowest WCSS). AIC and BIC were also used as additional criteria to validate k, and the final solution was evaluated using the amount of variance explained by the cluster solution as well as a silhouette score (ranging from -1 to 1 and describing the degree of cluster cohesion) with positive scores indicating better cluster cohesion and separation and negative scores or a score of zero suggesting no detectable cluster. Kmean analysis were conducted using base R (Team, 2013) k-mean function. The significance of the relationship between classification and cluster results were analysed using a chi-square test. Additional analysis and visualisation were conducted using JASP and FactoMiner functions (Lê, Josse, & Husson, 2008; Love et al., 2019), ggplot2 (Wickham & Wickham, 2016) and palates from RColorBrewer (Neuwirth & Brewer, 2014).

References

Kinner, V. L., Kuchinke, L., Dierolf, A. M., Merz, C. J., Otto, T., & Wolf, O. T. (2017). What our eyes tell us about feelings: Tracking pupillary responses during emotion regulation processes. *Psychophysiology*, *54*(4), 508–518. doi:10.1111/psyp.12816

Lang, P., & Bradley, M. M. (2007). The International Affective Picture System (IAPS) in the study of emotion and attention. *Handbook of Emotion Elicitation And*.

Lang, P. J., Bradley, M. M., & Cuthbert, B. N. (1997). International affective picture system (IAPS): Technical manual and affective ratings. *NIMH Center for the Study Of*.

Lê, S., Josse, J., & Husson, F. (2008). FactoMineR : An *R* package for multivariate analysis. *Journal of Statistical Software*, *25*(1). doi:10.18637/jss.v025.i01

Liaw, A., & Wiener, M. (2002). Classification and regression by randomForest. *R News*.

Love, J., Selker, R., Marsman, M., Jamil, T., Dropmann, D., Verhagen, J., … Wagenmakers, E.-J. (2019). jasp : graphical statistical software for common statistical designs. *Journal of Statistical Software*, *88*(2). doi:10.18637/jss.v088.i02

Neuwirth, E., & Brewer, R. C. (2014). ColorBrewer palettes. *R Package Version*.

Selker, R., Love, J., & Dropmann, D. (2020). jmv: The "jamovi" Analyses. *R package version*, *1*(2), 23.

Team, R. C. (2013). R: A language and environment for statistical computing.

Wickham, H., & Wickham, M. H. (2016). The ggplot package. *URL: Https://Cran. R*.
